# Flower-Shaped Carbon Nanomaterials for Highly Efficient Solar-Driven Water Evaporation

**DOI:** 10.3390/molecules27217163

**Published:** 2022-10-23

**Authors:** Nan Wang, Haifeng Xu, Jixin Yao, Bo Yang, Guang Li, Zhi Bai

**Affiliations:** 1 Key Laboratory of Spin Electron and Nanomaterials of Anhui Higher Education Institutes, School of Mchanical and Electronic Engineering, Suzhou University, Suzhou 234000, China; 2 School of Information Engineering, Suzhou University, Suzhou 234000, China; 3 Universities Joint Key Laboratory of Photoelectric Detection Science and Technology in Anhui Province, School of Electronic Information and Electrical Engineering, Hefei Normal University, Hefei 230601, China; 4 School of Physics and Electronic Information, Huaibei Normal University, Huaibei 235000, China; 5 Anhui Key Laboratory of Information Materials and Devices, Institute of Physical Science and Information Technology, School of Materials Science and Engineering, Anhui University, Hefei 230601, China; 6 School of Mechanical and Electronic Engineering, Suzhou University, Suzhou 234000, China

**Keywords:** solar-driven water evaporation, water purification, carbon nanomaterials

## Abstract

Solar-driven interface water evaporation is an energy-saving, environmentally friendly, and efficient seawater desalination and wastewater treatment technology. However, some challenges still restrict its further industrial development, such as its complex preparation, heavy metal pollution, and insufficient energy utilization. In this study, a photothermal layer based on flower-shaped carbon nanoparticles is presented for highly efficient solar-driven interface evaporation for water treatment applications. The results show that the surface of the prepared carbon nanomaterials presents a flower-shaped structure with an excellent light absorption capacity and a large specific surface area. Moreover, the C-5.4 (Carbon-5.4) sample has an evaporation rate of 1.87 kg/m^2^/h and an evaporation efficiency of 87%—far higher than most photothermal materials. In addition, carbon nanomaterials have an excellent ion scavenging capacity, dye purification capacity, and outdoor practical performance. This study provides a new solution for the application of carbon nanomaterials in the field of water purification.

## 1. Introduction

The freshwater resources on the earth account for only 0.3% of the total global water volume, and the freshwater resource crisis has become an important factor that limits the sustainable development of society [1,2]. With population growth, and increasing domestic wastewater, industrial pollution, and water wastage, the problem of freshwater resource shortage will be further aggravated; therefore, the desalination of seawater and the purification of wastewater technologies are of strategic importance in terms of solving the global scarcity of freshwater resources and achieving sustainable development. At present, multistage flash distillation [3,4], multieffect distillation [5,6], and reverse osmosis [7,8] technologies are widely used in the field of seawater desalination and wastewater purification and have achieved good results. However, there are also disadvantages, such as the ease of scaling, the high energy consumption, and the stability of filter membranes. Therefore, it is imperative to develop a new water evaporation system that is environmentally friendly, efficient, and economical for water purification.

Solar-driven interfacial water evaporation technology utilizes a photothermal material, floating at the gas–liquid interface, to capture solar energy and stay at the water evaporation interface. This not only reduces heat loss from the overall water heating by the photothermal material but also provides a larger surface area for the rapid release of vapor to achieve a higher evaporation efficiency [9,10,11,12]. Photothermal materials are the main factors affecting evaporation performance. A large number of studies have reported that various types of photothermal materials are used for solar interface water evaporation, mainly including carbon-based materials [13,14] and metal plasma materials [15,16,17].

Metal plasma materials are widely used in solar interface water evaporation due to their excellent light absorption performance. When light is incident on the precious metal nanoparticles, if the vibration frequency of the precious metal nanoparticles matches the incident photon frequency, the nanoparticles will strongly absorb the photon energy, thereby localizing the surface plasmon resonance phenomenon, thus improving the photothermal conversion efficiency [18]. Margeson et al. [19] loaded metal nanoparticles (Au and Ag) on superhydrophobic cotton to prepare Au/Ag-TDI-Cotton for use as a photothermal material. They verified its excellent water evaporation, desalination, and purification capabilities. Xu et al. [20] constructed an Ag/PPy photothermal layer and coated it on six different substrates. The cellulose fiber matrix film has an ultra-high solar evaporation rate of 1.55 kg/m^2^/h, a corresponding light vapor efficiency of 92.6%, and an obvious antibacterial effect. Chen et al. [21] decorated wooden flowers with hydrophilic and highly absorbent Ag-polydopamine core-shell-structured nanoparticles and improved the overall absorption capacity of the evaporator using the 3D architecture design of the flowers. The evaporation rate reached 2.08 kg/m^2^/h, and the evaporation efficiency reached 97%. Qian [18] prepared Au-CuS hybrid nanoparticles, which improved the water evaporation rate using its strong light absorption ability. Ren et al. [22] fabricated a self-propelled aerogel evaporator composed of the Schiff-base hydrogel and deposited Au layer. The water evaporation rate reached 3.12 kg/m^2^/h in natural seawater under 1 sun illumination. Furthermore, the self-healing property endowed by dynamic Schiff-base linkages and hydrogen bonds makes aerogel evaporators durable and stable. Most studies on solar water evaporation using plasma metal materials focus on metals such as gold, silver, and copper. However, the high cost and scarcity of gold and silver, and the fact that the metal nanoparticles and non-biodegradable synthetic polymers that are easily separated from the membrane structure may cause additional negative effects on the environment make their use problematic [23]. Therefore, finding efficient and environmentally friendly photothermal materials is vital.

Carbon-based materials have been studied for solar-driven water evaporation applications as they are environmentally friendly materials. Li et al. [24] and Wilson et al. [25] developed PDMS CNP solar absorbers with a 3D structure by embedding carbon nanoparticles into porous sponges, which not only have excellent water evaporation performance but also have good desalination performance in the floating state. Ye et al. [26] prepared superhydrophobic carbon black nanoparticles and suspended them on the water surface by molecular thermal movement and water tension to form CB films, which verified the excellent stability and durability of CB floating films and their good water evaporation performance. Meng et al. [27] prepared a double-layer solar evaporator with a coating of zeolitic imidazolate framework-8 (ZIF-8)-derived nanostructured carbon on a wood sponge for soil water extraction. Ming et al. [28] assembled two-dimensional MXene and GO into a 3D portable GMA water purification system, which can realize water purification and remove salt, organic pollutants, bacteria, and other pollutants quickly and effectively. Although these materials have achieved certain effects, GO, MXene, and carbon nanotubes are expensive and have a complex manufacturing process and carbonized biomass materials are simple and inefficient. Therefore, there is still a need to find a low-cost, durable, and efficient material with which to build a solar interface water evaporation system.

In this work, we used resorcinol as a precursor to prepare carbon particles with a flower-shaped structure as a light absorber, and hydrophilic cotton sheets were used as a substrate to increase the transport of water molecules. On this basis, we prepared a solar interface water evaporator to achieve continuous, efficient, and stable solar-driven interface evaporation. This evaporator has several main features: Firstly, the rough surface of the flower-shaped carbon particles can enhance the internal reflection of the incident light and thus enhance the absorption of incident light. Secondly, the porous structure on the surface of the carbon flowers can effectively pump water to the evaporation interface and improve the water transport speed. Thirdly, the evaporator is simple to prepare and sustainable. The experimental results show that the evaporation rate of all samples is higher than 1.5 kg/m^2^/h, and the C-5.4 sample performs best. Under a sunshine intensity of 1 kW/m^2^, the evaporator can reach an evaporation rate of 1.87 kg/m^2^/h and a photothermal conversion efficiency of 87%. Meanwhile, the evaporator has a highly efficient water purification capacity in practical applications.

## 2. Results and Discussion

### 2.1. Characterization

**Figure 1:**
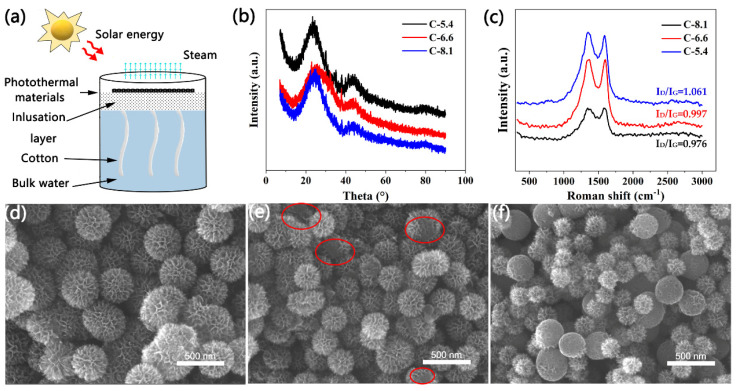
(**a**) Schematic illustration of the solar evaporation experimental device. (**b**) XRD patterns of C-5.4, C-6.6, and C-8.1 carbon nanomaterials. (**c**) Raman spectra of C-5.4, C-6.6, and C-8.1 samples. (**d**) SEM surface image of the C-5.4 carbon nanomaterials. (**e**) SEM surface image of the C-6.6 carbon nanomaterials. (**f**) SEM surface image of the C-8.1 carbon nanomaterials.

In order to explore the phase structure of the C-5.4, C-6.6, and C-8.1 samples, they were tested using XRD. It can be seen from Figure 1b that when the 2θ angle appears around 22° and 43°, a strong broad diffraction peak and a weak broad diffraction peak were observed, which correspond to the (002) and (100) diffraction peaks of graphite carbon, respectively, indicating that they have the characteristics of amorphous carbon. In the Raman spectrum of the C-5.4, C-6.6, and C-8.1 samples shown in Figure 1c, the two peaks of carbon nanomaterials were observed at approximately 1340 cm^−1^ (D band) and 1580 cm^−1^ (G band). The relative intensity ratios of the D peak to the G peak for C-5.4, C-6.6, and C-8.1 were 0.976, 0.997, and 1.06, respectively, indicating that the C-5.4 sample has more defects than the other two samples. The surface morphology of C-5.4, C-6.6, and C-8.1 carbon nanomaterials was observed using a scanning electron microscope (SEM). It can be seen from Figure 1d–f that the C-5.4 surface presented a flower-shaped structure with a uniform diameter, the average diameter of the carbon nanomaterials was 200 nm, and the C-6.6 surface presented the same flower-shaped structure, but the flower-shaped structure on some carbon particles was not completely displayed (marked in red). However, the C-8.1 sample had a large number of carbon particles without flower-shaped structures, and their surfaces were relatively flat.

**Figure 2:**
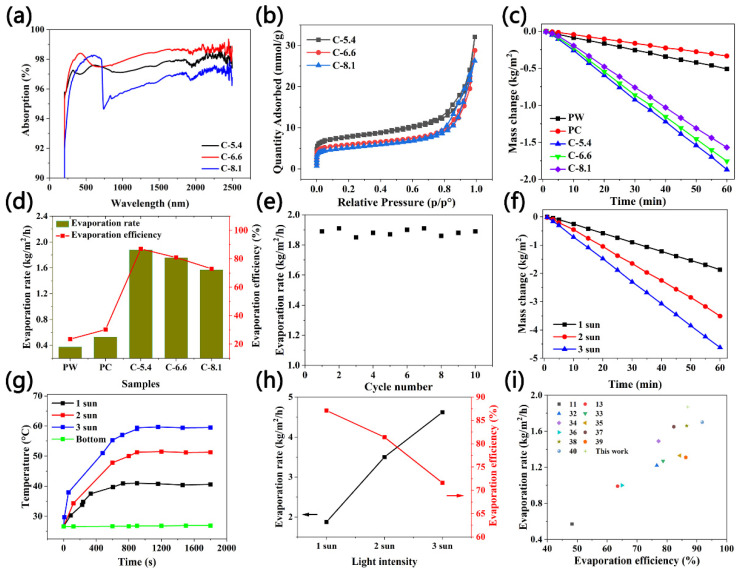
(**a**) Absorption spectra of C-5.4, C-6.6, and C-8.1 carbon nanomaterials. (**b**) Nitrogen adsorption/desorption isotherms. (**c**) The mass change in PW, PC, C-5.4, C-6.6, and C-8.1 samples under 1 sun. (**d**) The evaporation rate and efficiency of the PW, PC, C-5.4, C-6.6, and C-8.1 samples under 1 sun. (**e**) The cycling stability of the C-5.4 evaporator under 1 sun irradiation for 10-cycle evaporation measurements. (**f**) The mass change in the C-5.4 sample under 1, 2, and 3 sun. (**g**) The surface temperature change in the C-5.4 sample under 1, 2, and 3 sun, and the bottom temperature under 1 sun. (**h**) The evaporation rate and efficiency of the C-5.4 sample under 1, 2, and 3 sun. (**i**) The comparison of the evaporation rate and efficiency of the C-5.4 sample with those reported.

The light absorption capacity of carbon nanomaterials has an important impact on the evaporation performance of the system [29]. The light absorption of the C-5.4, C-6.6, and C-8.1 samples was measured using a UV-vis spectrophotometer. It can be seen from Figure 2a that, in the wavelength range of 200~2500 nm, the light absorption of the three samples exceeded 95%, while the light absorption of the C-5.4 sample was the highest (98.5%) and the C-8.1 sample was the lowest (96.5%). The high absorption efficiency results from both the high inherent absorption rate of carbon particles and the flower-shaped three-dimensional structure formed by carbon particles that limits light scattering [29,30]. The pore structures of the C-5.4, C-6.6, and C-8.1 samples were investigated by N_2_ adsorption–desorption at 77 K. The results are shown in Figure 2b. Obviously, the C-8.1 sample showed the highest N_2_ adsorption capacity, indicating a higher porosity and specific surface area. In addition, three samples showed type IV adsorption isotherms with obvious hysteresis loops, which indicates more micropores and mesopores. The specific surface areas of the C-5.4, C-6.6, and C-8.1 samples were 517.3087, 416.3272, and 371.9667 m^2^/g, respectively. Generally, the layered porous structure and relatively high specific surface area were conducive to light capture and water transmission [31].

### 2.2. Water Evaporation Performance

To measure the evaporation performance of the evaporator, the water was contained in a 250 mL beaker wrapped with PTFE, and the solar evaporation performances of the flower-shaped carbon nanomaterials (C-5.4, C-6.6, and C-8.1) were measured under 1 kW/m^2^ for 60 min. The water evaporation experiments on PW (pure water) and PC (pure cotton) were also carried out under the same conditions as the comparison group. 

In Figure 2c, there is the same downward trend in mass change over time under the 1 kW/m^2^ condition. However, the curve of the evaporator with carbon nanomaterial samples has a higher slope, indicating a higher mass loss at the same time as the control group and reflecting a superior solar–thermal energy conversion ability. It can be seen from Figure 2d that the evaporation rate of the PW sample was 0.375 kg/m^2^/h because the majority of energy from the xenon lamp was reflected and absorbed into the whole body of water. In addition, the evaporation rate of the PC sample is 0.527 kg/m^2^/h, a little higher than that of the PW sample. Obviously, the evaporation rate was greatly improved to 1.57–1.87 kg/m^2^/h by the flower-shaped carbon nanomaterial solar evaporators, which was much higher than those of the PW and PC samples, especially for the C-5.4 sample, which was five times that of the PW sample. This was due to the high light absorption performance and the thermal insulation layer reducing the heat transfer to the water. It can be seen that the evaporation rate decreased from 1.87 kg/m^2^/h to 1.57 kg/m^2^/h, with increasing ammonia from 5.4 mL to 8.1 mL. This indicated that the flower-shaped carbon nanomaterials have a good solar steam generation performance. The evaporation efficiency of the flower-shaped carbon samples was C-5.4 (87%), C-6.6 (80.9%), and C-8.1 (73%), which were higher than the evaporation efficiencies of the PC (30.2%) and PW (23.5%) samples. The decrease in the evaporation efficiency of the carbon nanomaterial samples was mainly due to the influence of the destroyed flower-shaped structures, which reduced the light absorption capacity and the water channel. Furthermore, Figure 2e demonstrates the cycling stability of the C-5.4 evaporator under 1 sun for 10-cycle evaporation measurements, with each cycle lasting for 60 min. The evaporation rate was basically maintained in the range of 1.86–1.90 kg/m^2^/h, indicating a good evaporation stability.

**Figure 3:**
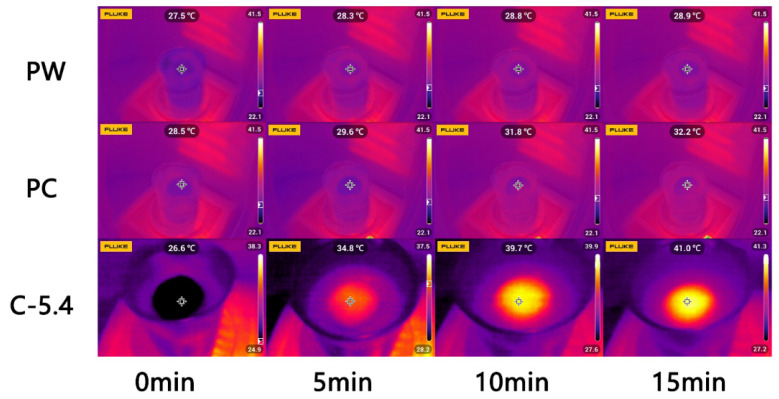
Infrared images of the PC, PW, and C-5.4 under 1 sun.

It can be seen from Figure 2f that the slope of the mass loss curve increases as the light intensity increases for the C-5.4 sample, indicating that more water evaporated at the same time. Figure 2h shows the evaporation rate of the C-5.4 sample under the solar light intensities of 1, 2, and 3 sun; the evaporation rate increases as the light intensities increased, and the evaporation rate corresponded to 3.51 kg/m^2^/h and 4.62 kg/m^2^/h when the light intensities were 2 and 3 sun. However, with the increase in light intensity, the evaporation efficiency decreased from 87% to 71.6%. Figure 3i shows the comparison of the evaporation rate and efficiency of the C-5.4 sample with those reported under 1 sun. It can be seen that the evaporation performance was better than the majority of those reported previously [11,13,32,33,34,35,36,37,38,39] but a little worse than that reported in the literature [40]. This is because, in this paper, we took into account the decrease in the evaporation enthalpy with the sample.

We further studied the solar-driven water evaporation performance of the C-5.4 sample, using the infrared thermal imager to measure the surface temperature. The surface temperature of the C-5.4 sample increased from 26.7 °C to 40.1 °C within 12 min and was stable at approximately 40 °C under 1 sun. It can be seen from Figure 2g that as the light intensity increased, the temperature growth rate was accelerated in the initial stage, and the steady-state temperature reached 51 °C and 60 °C when the light intensity increased to 2 and 3 sun, respectively. Although the surface temperature of the C-5.4 sample increased in the initial stage and then remained stable, the beaker bottom temperature was relatively stable, only rising 0.6 °C, indicating that the heat was absorbed by the upper layer photothermal materials. The comparison of the infrared images of the PW, PC, and C-5.4 samples under 1 sun is shown in Figure 3. The temperature variation of the PW sample was only 1.4 °C, which is less than those of the PC and CC samples, i.e., 3.3 °C and 14.4 °C, respectively.

### 2.3. Water Treatment Performance

**Figure 4:**
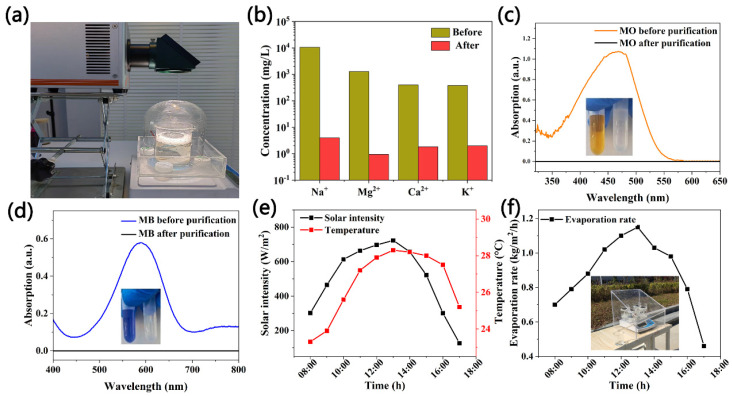
(**a**) Diagram of the freshwater collection device. (**b**) The concentration of Na^+^, Ca^2+^, K^+^, and Mg^2+^ in seawater before and after desalination. (**c**) Absorption spectrum of MO before and after purification. (**d**) Absorption spectrum of MB before and after purification. (**e**) Solar intensity and ambient environment in outdoor experiments. (**f**) Evaporation rate and equipment in outdoor experiment.

In order to verify the feasibility of these three flower-shaped carbon nanomaterials for seawater desalination and wastewater treatment, simulated seawater and prepared wastewater (methyl orange (MO) and methyl blue (MB)) were used in the solar-driven water purification treatment. Figure 4a shows the freshwater collection device used in the experiment. As a result of the use of transparent devices, it can maintain a full range of sunlight reception. Figure 4b shows that the concentrations of four primary ions (Na^+^, Mg^2^+, Ca^2^+, K^+^) were 10,620 mg/L, 1280 mg/L, 403 mg/L, and 380 mg/L in the simulated seawater, respectively. After the solar-driven desalination process, the concentrations of four primary ions (Na^+^, Mg^2^+, Ca^2^+, K^+^) sharply decreased, i.e., they were 4 mg/L, 0.95 mg/L, 1.83 mg/L, and 2 mg/L, respectively. The desalinated water after treatment was higher than the drinking water standard defined by the World Health Organization, which proves that the evaporator has a good desalination effect. For the wastewater purification experiment, MO and MB were selected to simulate the pollutants in dye wastewater. The collected samples were subjected to a UV-vis spectral analysis. As shown in Figure 4c,d, MO had an obvious absorption peak at 450 nm, and MB had an obvious absorption peak at 600 nm. However, no obvious absorption peak was found in the treated samples, and they became colorless. This shows that this evaporator can purify industrial wastewater represented by dyes.

In this study, we prepared a portable outdoor evaporator with a size of 30 × 30 × 30 cm (length × width × height) using transparent acrylic plates, as shown in Figure 4f. Under natural light, the steam generated by the air–water interface condenses on the surface of the upper slope, flows along the slope into the bottom tank, and then the freshwater flows into the collecting beaker through an outlet in the tank. The experiment was conducted in the open space of Suzhou University from 8:00 to 17:00 on 27 August 2022. Outdoor sunlight intensity and ambient temperature are shown in Figure 4e. During the test, we measured the mass loss every 1 h for 30 min each time, which represented the evaporation rate of the starting time. The results are shown in Figure 4f. It can be seen that the solar sunlight intensity ranged from 126.1 to 723.2 W/m^2^, and the maximum value appeared at around 13:00. Moreover, the average solar sunlight intensity between 8:00 and 17:00 was approximately 507 W/m^2^. The ambient temperature varied in the range of approximately 23.3 to 28.4 °C. The light intensity was basically synchronized with the ambient temperature. In addition, the mass loss of the C-5.4 sample was positively correlated with the light intensity, with a little decrease as compared to the room environment because the evaporated water vapor adhered to the inner wall of the cup cover causing light reflection [41].

## 3. Experimental

### 3.1. Materials

Resorcinol, formaldehyde, and ammonia were purchased from Macklin Inc. Sodium hydroxide was purchased from Fuchen (Tianjin) Chemical Reagent Co., Ltd. (Tianjin, China). Tetrapropyl orthosilicate was purchased from Picasso. Ethanol was purchased from Shanghai Titan Scientific Co., Ltd. Deionized water was used throughout the experiments. All the materials were used without further purification.

### 3.2. Fabrication of Carbon Particles

The details of the synthetic operation are as follows: (1) Four solutions containing deionized water (60 mL) and ethanol (180 mL) were each mixed and stirred well at room temperature for 5 min. (2) Totals of 5.4 mL, 6.6 mL, and 8.1 mL of ammonia with a concentration of 28% were added to the above four mixtures; these were marked as C-5.4, C-6.6, and C-8.1, respectively; stirring was continued at room temperature for 30 min. (3) A total of 1.8 g resorcinol and 2.4 mL formaldehyde were added to the above solutions in sequence; stirring was continued at room temperature for another 15 min, and then 15 mL tetrapropyl orthosilicate was added to the above four solutions before magnetic stirring at room temperature for 24 h. (5) The mixture was centrifuged and dried at 70 °C for 24 h in the oven. (6) The resulting solution was carbonized at 850 °C for 2 h in an argon atmosphere and naturally cooled to room temperature. (7) It was placed in sodium hydroxide solution for 24 h before washing to neutral.

### 3.3. Characterization of Samples

The morphological structure was observed using a scanning electron microscope (TESCAN MIRA LMS, Brno, Czech Republic). An X-ray diffractometer (ZSX PrimusIV, Rigaku Corporation, Tokyo, Japan) was used to measure the X-ray diffraction pattern with a scanning rate of 2°/min, a 40 kW voltage, and a 50 mA current. A UV-Vis spectrophotometer (Lambda750s, Perkin Elmer instruments (Shanghai) Co., Ltd., Shanghai, China) was used to measure the light absorption performance. A physical adsorption instrument (ASAP 2020 Plus HD88, Micromeritics, Norcross, GA, USA) was used to test the isothermal adsorption and desorption analysis of carbon materials. Inductively coupled plasma (PQ 9000, Analytik Jena AG, Jena, Germany) was used to test the concentrations of salt ions for the simulated seawater before and after evaporation. A Raman spectrometer (XPLORA PLUS, HORIBA Jobin Yvon, Paris, France) was used to test the structural characteristics of carbon materials.

### 3.4. Solar Water Evaporation Experiment

The principle of solar-driven water evaporation technology is to use solar energy (or a simulated light source) to heat the photothermal material. The heat is then transferred to the surface water below the material (instead of heating the water body as a whole) to make it evaporate and escape. Thus, water channels continuously transport water to the photothermal layer to ensure evaporation continues. Finally, it is condensed and collected by the collector to produce fresh water. The experimental device is shown in Figure 1a. It mainly includes four parts: a light source, the photothermal material layer, the insulation layer, and the water channel. Polyethylene foam is used as a thermal insulation layer to isolate the heat from the light source transfer to the whole body of water. The prepared carbon materials are used for absorbing light from the light source. As a result of its good water absorption, the cotton column simulates the water channel, passing through the polyethylene foam under the carbon materials layer. The experiment mainly included a light source, a quality acquisition system, and a temperature acquisition system. A xenon lamp (CEL-HXF300-T3, CEAULIGHT, Beijing, China) was used to simulate sunlight, an electronic balance (JS-A5, CEAULIGHT, China) connected to a computer was used to measure the mass change of water, and an infrared thermal imager (PTi120, Fluke, Shanghai, China) was used to measure the surface temperature and take infrared images.

### 3.5. Solar Water Evaporation Performance Evaluation

Solar evaporation efficiency is defined as the ratio of the energy used for water evaporation to the radiated solar energy, as shown in Formulae (1) and (2) [42,43].

(1) $$\eta =\frac{v\cdot h_{e}}{C_{opt}\cdot q_{i}}$$

(2) $$he=\frac{h_{0}\cdot v_{0}}{v_{e}}$$

where *h_e_* and h_0_ refer to the total enthalpy change in the liquid–gas phase transition of the pure water and sample water, respectively (kJ/kg); *Copt* refers to the condensing coefficient; *q_i_* refers to one solar radiation intensity (1 kW/m^2^); and *v*_0_ and *v_e_* refer to the evaporation rate of the pure water and sample water in the dark environment, respectively, (kg/m^2^/h), which can be calculated according to Formula (3).

*v* = (△*m*)/(*S*⋅*t*) (3)

where △*m* refers to the total mass change during the test (kg); *t* refers to the corresponding evaporation time (h); and *S* refers to the evaporation area (m^2^).

## 4. Conclusions

In summary, a flower-shaped carbon nanomaterial was prepared for a solar evaporator for desalination and water purification application. The special surface structure greatly enlarges the specific surface areas of carbon nanomaterials and improves the light absorption capacity. As a result, the carbon-based evaporator achieves an efficient and stable water evaporation rate of 1.87 kg/m^2^/h and an evaporation efficiency of 87% under 1 sun, which is superior to the carbon materials in the literature. In addition, the evaporator based on the carbon nanomaterial shows outstanding removal capabilities for salt ions and dye molecules, as well as practicability in outdoor environments. This work provides an efficient but simple method for the fabrication of high-performance solar-driven water evaporation materials and devices for seawater desalination and wastewater purification.

## Data Availability

Data available from the corresponding author.
